# Enhancing Mitigation of Volumetric DDoS Attacks: A Hybrid FPGA/Software Filtering Datapath

**DOI:** 10.3390/s23177636

**Published:** 2023-09-03

**Authors:** Denis Salopek, Miljenko Mikuc

**Affiliations:** Faculty of Electrical Engineering and Computing, University of Zagreb, 10000 Zagreb, Croatia; miljenko.mikuc@fer.hr

**Keywords:** hybrid filters, DDoS mitigation, low power, FPGA, hardware/software packet processors, high performance

## Abstract

The increasing network speeds of today’s Internet require high-performance, high-throughput network devices. However, the lack of affordable, flexible, and readily available devices poses a challenge for packet classification and filtering. This problem is exacerbated by the increase in volumetric Distributed Denial-of-Service (DDoS) attacks, which require efficient packet processing and filtering. To meet the demands of high-speed networks and configurable network processing devices, this paper investigates a hybrid hardware/software packet filter prototype that combines reconfigurable FPGA technology and high-speed software filtering on commodity hardware. It uses a novel approach that offloads filtering rules to the hardware and employs a Longest Prefix Matching (LPM) algorithm and allowlists/blocklists based on millions of IP prefixes. The hybrid filter demonstrates improvements over software-only filtering, achieving performance gains of nearly 30%, depending on the rulesets, offloading methods, and traffic types. The significance of this research lies in developing a cost-effective alternative to more-expensive or less-effective filters, providing high-speed DDoS packet filtering for IPv4 traffic, as it still dominates over IPv6. Deploying these filters on commodity hardware at the edge of the network can mitigate the impact of DDoS attacks on protected networks, enhancing the security of all devices on the network, including Internet of Things (IoT) devices.

## 1. Introduction

The rapid growth of the Internet, amplified by the proliferation of Internet of Things (IoT) devices and coupled with increasing network speeds, requires the deployment of high-performance, high-throughput network devices that prioritize energy-efficient solutions. In the context of IoT environments, the challenge lies in developing efficient mechanisms that can process packets rapidly while optimizing energy usage to meet their energy-constrained demands, even at the network edge.

Packet processing in high-throughput networks is primarily the task of specialized hardware-based network devices that can quickly classify and filter packets, albeit with limitations. Performing this task programmatically on a 100 Gbps network requires packet filtering that can handle a throughput of over 148 million packets per second (Mpps). On a general-purpose computer with a processor speed of 4 GHz, this would mean that each packet should be processed in less than 27 clock cycles.

Depending on the type of device, the classification of packets is based on certain criteria, such as searching by destination MAC address or the VLAN tag in switches, or the source/destination IP address in routers and firewalls. Performing such checks within the required 27 clock cycles is a major challenge, as multiple operations or memory fetches must be completed within this time frame. The 27 clock cycle example applies to the smallest frame size traffic, when the device is at its busiest due to the shortest packet arrival times. Regardless of this, devices in the network infrastructure must be able to handle packets of all sizes at all speeds. Otherwise, if one device fails, its availability can no longer be guaranteed.

Malicious users on the Internet exploit this fact and attempt to disable access to certain services through Denial of Service (DoS) attacks and Distributed Denial of Service (DDoS) attacks. According to various sources [1,2,3], DDoS attacks are becoming increasingly common. In these attacks, infected computers under the control of attackers (called *bots*, often insecure computers or IoT devices) send traffic to the victim, consuming resources and disrupting normal users. Defending against such attacks is very difficult, as there may be millions of these infected devices. Distinguishing the ‘good’ traffic from the ‘bad’ and at the same time filtering it out is particularly problematic when dealing with very high network speeds.

Despite current efforts to replace IPv4 with IPv6 in response to IPv4 address exhaustion, there is no clear indication that this transition will occur in the near future. Since the percentage of total IPv6 traffic remains lower [4], the occurrence of IPv6 DDoS attacks is also relatively limited. Therefore, this paper focuses exclusively on IPv4 traffic and the mitigation of IPv4 DDoS attacks, as it is expected that IPv4 will still be in use for an extended period of time.

It is important to differentiate between DDoS protection (mitigation) and DDoS detection (recognition). DDoS mitigation systems may include DDoS detection, but this is not universally the case. This paper focuses primarily on packet filtering and the mitigation of detected DDoS attacks, assuming that other systems are responsible for the automatic or non-automatic task of DDoS detection.

The work is organized as follows. Section 2 gives an overview of related work in the protection against DDoS attacks. Section 3 describes a model of a hybrid hardware/software datapath used for high-speed packet filtering. Section 4 explains the benchmark methodology and showcases the results of the hybrid filter compared to the software-only filter. Finally, Section 5 provides the conclusion.

## 2. Related Work

The current state of protection against DDoS attacks relies on using one of three types of protection approaches: third-party delegation, on-site infrastructure protection, or a combination of both. Third-party delegation routes all traffic to a DDoS protection service, which then “scrubs” the dangerous and suspicious traffic as needed and redirects legitimate traffic to its destination. However, this redirection of traffic raises potential issues if the traffic is sensitive to even minor delays or contains sensitive and private information that third parties should not have access to (e.g., in the financial sector).

On-site protection is achieved by devices capable of filtering traffic using specialized hardware, software, or a hybrid of both. Hardware-based filtering is performed by devices designed specifically for this purpose. These devices offer high throughput, but come with high annual licensing costs for the associated software. Apart from the cost, negative aspects of such devices include inflexibility and complexity when it comes to modifications or updates [5]; so, after a few years, they no longer meet the requirements of current network speeds. In addition, the use of primarily TCAM (ternary content-addressable memory) technology in these devices contributes to their high power consumption [5,6], which exacerbates their disadvantages. Other technologies used for this type of filtering include ASIC (application-specific integrated circuit) and FPGAs (field-programmable gate arrays), with ASIC having similar disadvantages to TCAM, including the high price of its development, but FPGAs standing out from both of them by being reprogrammable.

Software frameworks for fast packet processing on general-purpose computers have emerged in recent years. These frameworks, such as Netmap [7], DPDK [8], and XDP/eBPF [9], when combined with sufficiently adequate hardware, can achieve packet processing results comparable to hardware filters. They provide flexibility and control over the filters created with them because they are simpler and programmable compared to most hardware-based systems. Software-defined networking (SDN) using OpenFlow [10] and content delivery networks (CDNs) [11] have also been explored, but encounter architectural limitations [12] for the mitigation of large volumetric DDoS attacks [13,14].

Hybrid protection combines hardware- and software-based protection, often utilizing some form of hardware to partially (or fully) handle the filtering process and “offload” software-based filtering, which is expected to have lower performance. This is why hybrid hardware/software solutions combining software with non-expensive, off-the-shelf hardware (e.g., FPGAs [15,16,17,18,19,20], GPUs [21,22,23,24], or smart NICs [25,26,27]) offer a flexible and cost-effective approach.

In [15], packet processing is performed using eBPF, with its implementation running entirely on FPGAs, while [16] describes a framework for offloading click router functionality that also runs entirely on FPGAs. In these systems, the CPU is primarily used for system preparation and transport to the hardware component, and so there is no “hybridity” in the packet processing itself. However, in [17,20], offloading is achieved by moving a limited number of filtering rules to the FPGA, while the remaining rules are executed on the host machine using the Linux firewall. This reduces the load on the CPU due to the smaller number of rules on the host. The work of [18] and its improved version [19] achieve offloading in a similar manner, but for higher traffic throughput, along with improvements related to storing rules on the FPGA. The filtering method in all of these mentioned systems has limitations on the number of rules that can be offloaded, depending on the implementation and the resources available on the FPGA. This makes them inconvenient for defending against large volumetric DDoS attacks. In addition, modifying rules on the FPGA requires re-synthesizing the bitfile in the worst case, which is time-consuming and does not provide a fast defense in case of a DDoS attack.

In [21], the authors present ways to improve CPU packet processing using some principles of GPU processing (e.g., latency hiding). This work’s actual contribution is that the CPU is capable of processing packets faster than the GPU in certain cases, without the negative consequences associated with GPUs. The systems in [22,23,24] perform packet operations entirely on the GPU, i.e., they utilize the parallelism of the GPU in different ways to process a large number of packets simultaneously. In this way, the GPU effectively acts as a large number of CPUs. A similar mode of operation is found in [25], where all “heavy” packet processing is conducted on the GPU, while simpler tasks are left for processing on the CPU. All GPU-assisted hybrid solutions face latency issues due to the batch processing of packets and may introduce packet re-ordering. They are also not compatible with all GPUs and are less energy-efficient than FPGAs.

The system in [26], like some of those previously mentioned, involves transferring a certain number of rules to the (memory-limited) SmartNIC, while leaving the rest on the host computer. An approach to packet processing similar to our paper can be found in [27,28]. They also utilize some method of preprocessing of packets (generating metadata) that takes place before the actual packet processing on the CPU. However, these papers do not address cases involving defense against large volumetric DDoS attacks and the challenges associated with them. Instead, they use SmartNICs for packet preprocessing in conjunction with other applications (e.g., key–value stores or GRE termination).

Protection against DDoS attacks is approached in a similar way to a standard firewall, where rulelists are created with various fields to be checked (e.g., source or destination IP address, transport layer protocol, or ports) and each packet traverses these lists, comparing its header against the specified fields. In some cases, attempts are made to minimize these rulelists, or Packet Classification Engines (PCEs) are used to achieve a filtering method that requires minimal memory fetches. However, these tools work under the premise that protection against DDoS attacks is only possible with a large number of separate rules, requiring tens of thousands or even millions of such records.

To assess the effectiveness of DDoS filtering solutions, it is important to consider the type and volume of traffic used in testing. Any solution should be exposed to traffic that mirrors real DDoS attacks. A simulation of the attack can be achieved by flooding the filter with synthetic traffic containing randomly generated IP addresses or using existing traces of actual DDoS attacks. Some vendors [29,30] utilize genuine DDoS traffic, as they have access to extensive real-world data and traffic with various DDoS attack scenarios. However, since most researchers do not have access to such data, they resort to synthetic traffic to simulate DDoS attacks in their tests [18,19,26,31,32]. Synthetic traffic with a large pool of randomly generated IP addresses can approximate DDoS attacks, but in previously mentioned works, this is limited to tens of thousands of IP addresses and is therefore not capable of replicating the scale of today’s volumetric DDoS attacks. For example, the attack on Dyn in 2016 involved tens of millions of different IP addresses, as shown through various analyses [33,34,35]. Therefore, any effective DDoS filtering system must be able to withstand such large-scale attacks.

In addition to the “active” defenses against DDoS attacks mentioned above, it is worth mentioning so-called blackhole routing. In this method, the victim’s IP address is reported to the network service provider, which then redirects all traffic destined for that IP address to a “black hole”, effectively discarding it. This protects the rest of the network by saving bandwidth by eliminating a significant portion of malicious traffic, while effectively fulfilling the attackers’ goal by rendering the victim inaccessible to other users.

## 3. Hybrid System Model

This paper, based on the research from one of the author’s previous work [36], builds upon our previous studies [37] which aimed to enhance existing software-based filtering to protect against volumetric DDoS attacks by replacing large rulelists with more compact ones. At the same time, additional tables are used to store IP addresses or subnets (e.g., whitelists or blacklists), and so these can be retrieved much faster using Longest Prefix Matching (LPM). In this paper, we propose a hybrid filtering system using a combination of FPGA hardware and software support based on the aforementioned LPM filtering. This moves away from the paradigm that defense against DDoS attacks requires the maintenance of monolithic lists with complex rules.

The packets to be filtered first pass through the hardware, which parses them and, if necessary, generates metadata that are passed to the software filter. The software filter receives the packets that are then metadata-enriched and, depending on its programming, parses them and performs the required actions which are determined from the ruleset given to the system, explained later in this chapter.

When using allowlists/blocklists for filtering, the LPM algorithm plays a crucial role in extracting data from each list to determine whether the IP address being checked belongs to a specific list. Various LPM algorithms can be used for this task, but the current implementation of the filter uses the DXR algorithm from [38,39], specifically the D16X4R version.

The DXR algorithm compresses and stores a list in compact structures with a small memory footprint. The algorithm consists of three stages (D16, X4, and R), all originally executed in software. However, in the hybrid implementation, a modified version of the algorithm is used. The first two stages (D16 and X4—indexing and memory retrieval using a total of 20 bits) are separated from the last one and executed in hardware. Then, the hardware passes the result of these two stages (the 32-bit index and a range included in the metadata of the packet) to the software. In the last stage of the algorithm, a binary search is performed over the received range (R—range lookup) until it reaches its end and returns the final result representing the “next-hop” for a given LPM table or a null value, indicating no match.

To select the best ruleset/metadata combination depending on pre-specified assumptions, parameters, and factors, we heuristically modeled a distributor part of the hybrid system. It acts as an intermediary and determines how packets are parsed in hardware, what metadata are created, and which filter rules are appropriate for hardware offloading. It takes into account various parameters such as the ruleset, hardware, and software capabilities, network status, and traffic volume.

### 3.1. Hardware

FPGA technology was chosen for the hardware component due to its flexibility in terms of reconfiguration and the utilization of parallelism. This also made the FPGA a promising technology to improve the efficiency of packet processing in IoT environments [40]. NetFPGA SUME [41] is a development board for prototyping network functions for high-speed networks that has been used extensively in various research projects since 2015 [42,43,44,45,46,47]. It provides prototyping capabilities for such a filter at 10 G network speeds and features a Xilinx Virtex-7 690T FPGA, four 10 GbE SFP+ interfaces, QDR II SRAM memory modules, DDR3 SODIMM memory modules, and other peripherals.

**Figure 1:**
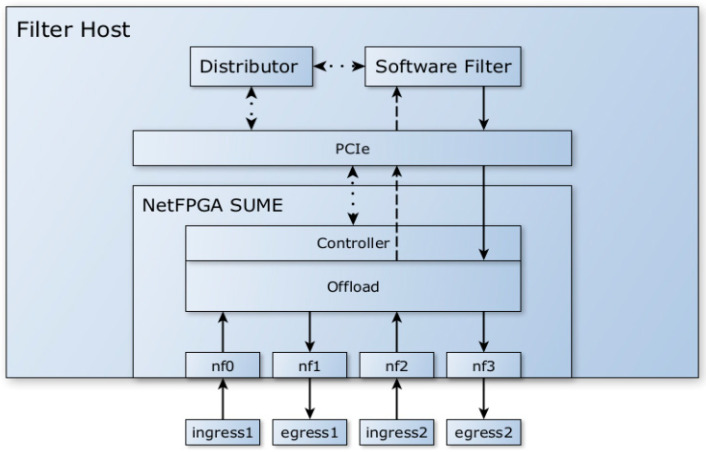
Architecture of the proposed DDoS filtering system using NetFPGA SUME NIC. Regular arrows represent “real” packet datapaths, dashed arrows represent combined “real” packet and metadata datapaths, and dotted arrows represent internal communication between different modules of the system.

The primary idea for our model was to configure NetFPGA SUME as an NIC on top of which the software filter would be deployed, and so it would serve as a standalone network middleware element installed at the edge of the network, as shown in Figure 1. The DMA engine used in the existing NetFPGA SUME Reference NIC project [48] was not designed to fully utilize the PCIe bus, and so the bandwidth between the NetFPGA SUME card and the operating system was poor. Attempting to create and implement an improved version which would work in a high-speed environment on the existing hardware proved impossible without significant and complex modifications.

**Figure 2:**
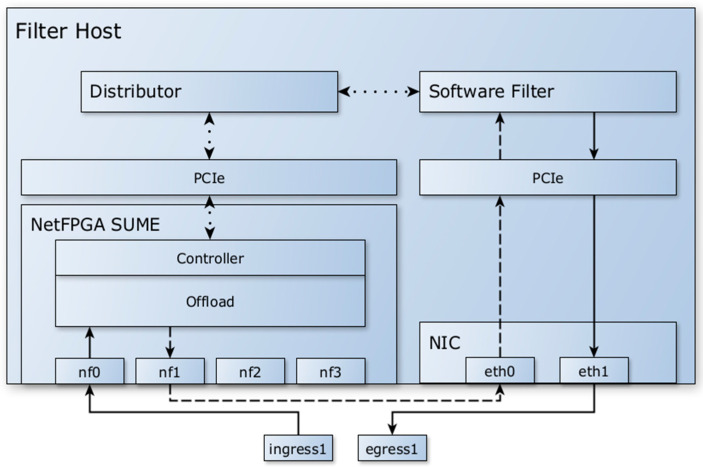
Architecture of the implemented DDoS filtering system without using NetFPGA SUME NIC. Regular arrows represent “real” packet datapaths, dashed arrows represent combined “real” packet and metadata datapaths, and dotted arrows represent internal communication between different modules of the system.

Therefore, the model was modified to no longer use the PCIe bus for packet transmission and to exclusively use Ethernet communication between the FPGA and the software filter. Packets and metadata are passed from the FPGA to the software filter via Ethernet, achieving sufficiently high speeds for use in 10 G networks. The model, as shown in Figure 2, uses an additional NIC to receive packets with metadata and forwards them to the software filter. The NetFPGA SUME performs the necessary offload and pre-filtering tasks, but the automatic forwarding of packets to the egress interface from hardware is disabled. The model can be extended by enabling additional interface pairs to increase the overall throughput.

The hardware implementation demonstrates the hybrid filter prototype datapath on the NetFPGA SUME development board, using the AXI4-Stream protocol for inter-module communication in the system pipeline. The pipeline is composed of modules connected in series or parallel and consists of two parts: one part carries packets arriving from the incoming network interface, i.e., packets that are checked (filtered) and forwarded to the output interface if necessary, and control packets from the “Distributor” that regulate the internal logic within the hardware (e.g., setting memory values or enabling and disabling certain parsers). All data required for filtering (e.g., source and destination IP addresses) are extracted from the “real” packets, and metadata are created based on this information. The metadata are appended to the end of the packets and forwarded to the software filter.

Two types of memory modules are used in the implementation: Block Random Access Memory (BRAM) and Quad Data Rate Static Random Access Memory (QDR SRAM). BRAM is a memory integrated on the FPGA board with limited capacity and very low latency (readout requires up to two clock cycles), while QDR is an external memory module with a larger capacity but slightly higher latency (about 20 clock cycles to readout). Both types of memory are suitable for high-speed operation, which is why they are used for packet filtering. In particular, they store the data required for the partial execution of the LPM algorithm, or other partially offloadable rule patterns which the hardware sends to the software filter in the metadata.

### 3.2. Software

The proposed system utilizes the Restricted Feature-set Packet Filter (RFPF)—a software filter developed in our previous research [36]. RFPF is a high-performance IPv4 traffic filter proven to be capable of filtering DDoS traffic at 10 G speeds using only one CPU core. It works by binding to two network interfaces using the Netmap software framework and generates C code from a predefined rulelist. The generated code is converted into a dynamically executable program that is inserted between the network interfaces to filter traffic in both directions. In this work, it is adapted to the hybrid mode of operation, considering how the filtering is performed in hardware and how the information from the metadata are used in the software filtering. The software filter separates the metadata arriving from the hardware from the packets themselves and uses them for further processing.

### 3.3. Rule Categorization

The rulesets used in the model consist of rules which are grouped and categorized by whether they are filtered in hardware, software, or both components. The rules consist of one *action* and one or more *patterns*, in the following format:

*action pattern {pattern…}*

The action can be *terminating* (if further packet inspection is halted after the rule is matched, e.g., *A*—Accept or *D*—Deny) or *non-terminating* (*N*—if the rule matching is continued even though the rule is matched). Additionally, the action may have a *counting* (*c*—when the software component must be notified that the rule was matched) or *non-counting* (*n*—if the rule does not require incrementing a counter) attribute. Patterns are divided into three types: those that can be fully or partially processed in hardware (p_O_—fully offloadable and p_P_—partially offloadable) and those that cannot be processed in hardware (p_N_—non-offloadable). The combination of patterns in a rule determines the overall rule *offloadability* attribute: fully offloadable (*O*), partially offloadable type 0 (*P*_0_), partially offloadable type 1 (*P*_1_), partially offloadable type 2 (*P*_2_), and non-offloadable (*N*), as shown in Table 1.

Combining the pattern *offloadability* attributes (five options), *terminating* attributes (three options), and *counting* attributes (two options), there are a total of 30 possible combinations of rule types that can be categorized by how they can be processed in hardware: fully, partially, or not at all. In the context of DDoS protection used in this research, *non-terminating* actions with the *non-counting* attribute make no sense, regardless of the pattern *offloadability* (*Nn); therefore, they are not considered. Table 2 presents the remaining 25 combinations, categorized based on the offload type that they belong to.

**Figure 3:**
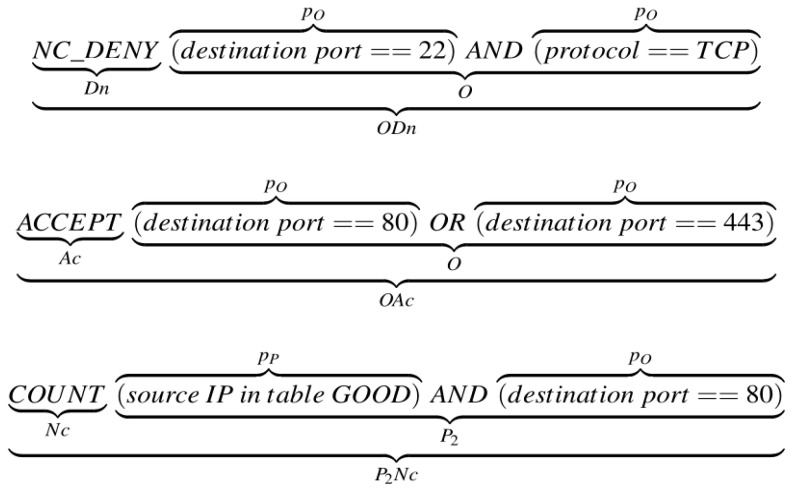
Three types of rules, annotated with their categories. The first rule specifies that every TCP packet with destination port 22 should be dropped and not counted (Deny with *non-counting* attribute). The second specifies that every packet with destination port 80 or 443 should be forwarded and counted (Accept with *counting* attribute). The first two rules are *terminating*—the filter stops parsing any subsequent rules if this rule is matched. The third rule is *non-terminating*. It specifies that packets with the source IP address from the GOOD table and with destination port 80 should be counted without any action—if there are rules after this one, they are checked.

An example of a pseudo ruleset with several different rule types categorized accordingly is shown and explained in Figure 3.

When taking into consideration the information to be exchanged between the hardware and the software during the filtering process, the categories can be classified into eight groups, with each group utilizing one of the four different metadata types, as demonstrated in Table 3:

- *metadata1*—data used in partially offloaded processing (e.g., protocol type, IP address source/destination, port number, or partial data used for an LPM algorithm).
- *metadata2*—data used in fully offloaded processing (a binary value for every p_0_ pattern from partially offloaded rules, whether the hardware processing matched the rule or it did not).
- *metadata3*—data used for all *counting* rules (a binary value for every *counting* rule, whether the hardware processing matched the rule or it did not).
- *metadata4*—data used when a *terminating* rule is matched (8-bit data noting the rule number that first matched).

As shown in Table 3, fully offloadable rules with the “Deny” *terminating* attribute and no *counting* attribute (*ODn*) can be offloaded to hardware without sending metadata to software exclusively if they appear at the beginning of the ruleset. Otherwise, the result of their check must be sent to the software using the same metadata as the fully offloadable rules with the “Accept” *terminating* attribute and no *counting* attribute (*OAn*). However, the implementation architecture used for this filter limits the inclusion of these two categories (*ODn* and *OAn*) in the performance evaluation presented in this paper.

The evaluation is solely conducted for all of the other groups of rules (except for N** group rules, which are non-offloadable by default). The matching of the fully offloadable *non-terminating* rules with the *counting* attribute (*ONc*) should only increment the counter in the software, and so they require one bit for each rule that can be counted. Fully offloadable rules with both *terminating* and *counting* attributes (*O*[*A*|*D*]*c*) need to send only the ordinal number of the first *terminating* rule that matches in hardware. For each combination of partially offloadable *P*_0_ rules, only the results of each fully offloadable pattern (p_O_) need to be sent to software as a true/false bitmap. For every combination of partially offloadable *P*_1_ rules, the hardware computes the data for partially offloadable patterns (p_P_) and sends it in full to the software. *P*_2_ rules have the same metadata requirements as *P*_0_ and *P*_1_ rules combined.

### 3.4. Use Case

**Figure 4:**
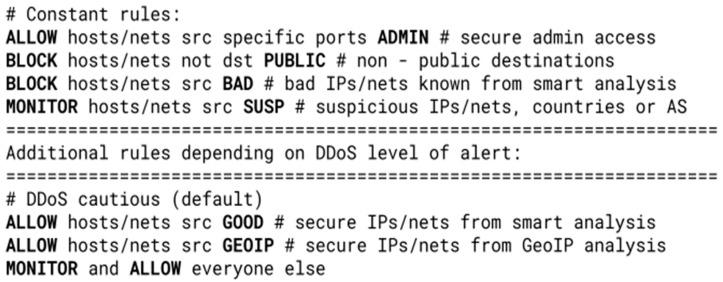
Pseudo ruleset example for *default* security status. The ‘#’ characters denote comments.

Combined with an external DDoS detection system, the filtering system described in this paper would effectively utilize the LPM search for IP addresses and subnets against DDoS attacks even with millions of different attackers in a high-speed networking environment. As an example, this kind of mitigation could be achieved using only seven rules with six different tables (lists of IP addresses and subnets), as shown in Figure 4. As the system is reconfigurable, various versions of rulesets could be made ready to be deployed depending on the security status of the network.

All such rulesets include constant rules that always perform the same tasks, regardless of the security level of alert, as well as variable ones that change depending on the security status. The rules from Figure 4 allow certain safe source hosts/networks to all necessary parts of the internal network, possibly further specified by destination ports (ADMIN table—e.g., third-party administrators that should always have access to the devices in the network). Access is blocked to all other parts of the network that are not publicly accessible (PUBLIC table—e.g., IP addresses of private email servers or IoT sensors) and to known malicious IP addresses (BAD table—e.g., from publicly available collectors). Additionally, traffic considered to be suspicious is exclusively monitored (SUSP table—e.g., subnet ranges from regions known for espionage or DDoS attacks).

The rest of the ruleset depends on the situation and may change depending on whether the network is under a DDoS attack and how severe it is. In the state of normal network activity (i.e., without DDoS attacks), an external tool that monitors regular traffic accumulates secure hosts/networks in a secure table that is always forwarded (GOOD table—e.g., regular or unsuspicious users). Using LPM also allows for the fast classification of source addresses according to their geo-location, and so it always forwards specific countries (GEOIP table—country for which the service is intended, neighboring countries, or “friendly” countries). All other traffic is forwarded, but also monitored by an external automatic DDoS attack detection system.

**Figure 5:**
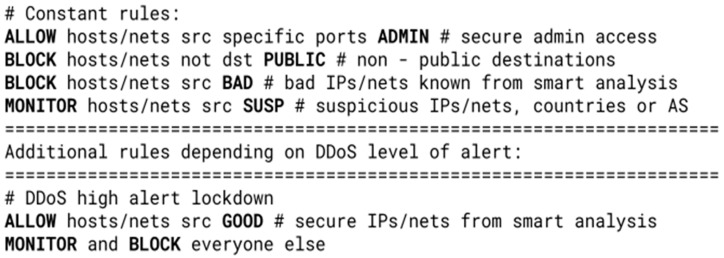
Pseudo ruleset example for *high*-*alert* security status. The ‘#’ characters denote comments.

During the DDoS attack, instead of allowing all unknown traffic from the variable part of the ruleset, the filter blocks everything except potentially secure tables (GOOD and GEOIP). Since all other traffic is monitored, this helps to isolate the bad IP addresses and adds them to the BAD table, which is updated accordingly. If necessary, the filter would additionally reject the GEOIP table, as shown in Figure 5, if it is proven to be unsafe, until the attack subsides or all the attackers are blocked.

## 4. Benchmarks

Since the packets in the implementation of this filter are prepared independently by hardware, with their metadata created before reaching the input interface of the software component, the software is unaffected by how they were created.

**Figure 6:**
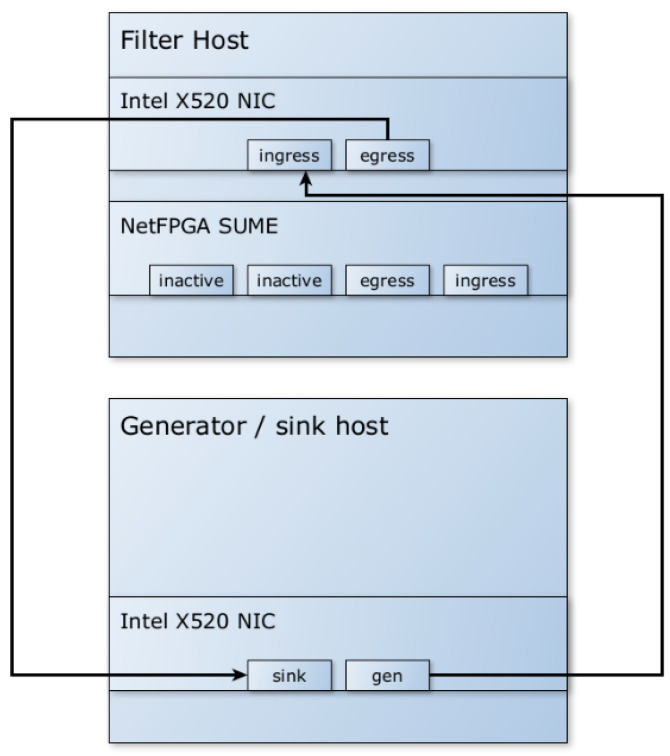
Simulated hardware testbed (bypassing the NetFPGA SUME).

To test and validate the system without the need for multiple hardware implementations, the measurements were designed to ensure the independence of packet preparation and metadata creation. This was achieved by simulating the hardware part of the system by creating the metadata programmatically. The *pkt-gen* tool (included in the *Netmap* framework) was used on a separate computer to generate packets, create the necessary metadata, attach it to the packets, and send them to the software filter, as shown in Figure 6. The filtered traffic is verified in the traffic sink, which also calculates the throughput of incoming packets.

The test results are based on the 60 s average, and the CPU cycle counter in the software filter is implemented using the assembler instruction *rdtsc* [49], which acquires the processor’s timestamp counter before and after processing each batch of packets.

To validate and verify the results of the simulated measurements, some of the measurements were performed on a real hybrid system that used FPGA hardware to generate metadata.

### 4.1. Results

We present a comparison of filtering results with and without offloading on hardware, using different hardware offloading configurations. The average total throughput and the average number of CPU cycles per packet were measured, and two types of measurements were performed: using *random* and *specific* traffic. Both types used randomly generated traffic with either completely random source IP addresses or specifically shaped traffic to seem like a DDoS attack. The *specific* traffic consisted of a combination of “normal” random traffic and randomly selected IP addresses from a large pool of malicious IP addresses. In this way, we could demonstrate how the filter performed under pressure, when the filtering load was high. All of the tests were set so that the bandwidth never reached the maximum possible value for the system. Additionally, to allow for better control and consistency of tests, all of the tests were performed on a system with a single CPU core at reduced frequency. For this reason, the efficiency of the two filtering methods could be compared based on the number of packets processed per second and the number of CPU cycles required to process one packet.

The measurements were categorized by rulelists, which were tested with associated metadata specific to the rules within them. Multiple measurements were conducted for each rulelist to obtain average results for all of the hardware offloading configurations: without any hardware offloading (only the software filter without metadata) as a point of reference, and then with modified parameters for hardware offloading. Each individual rulelist consisted of multiple rules, created in such a way to test the offloading of a single metadata type and, in some cases, combinations of multiple types of metadata. The detailed explanations and extended results of these experiments can be found in [36].

**Figure 7:**
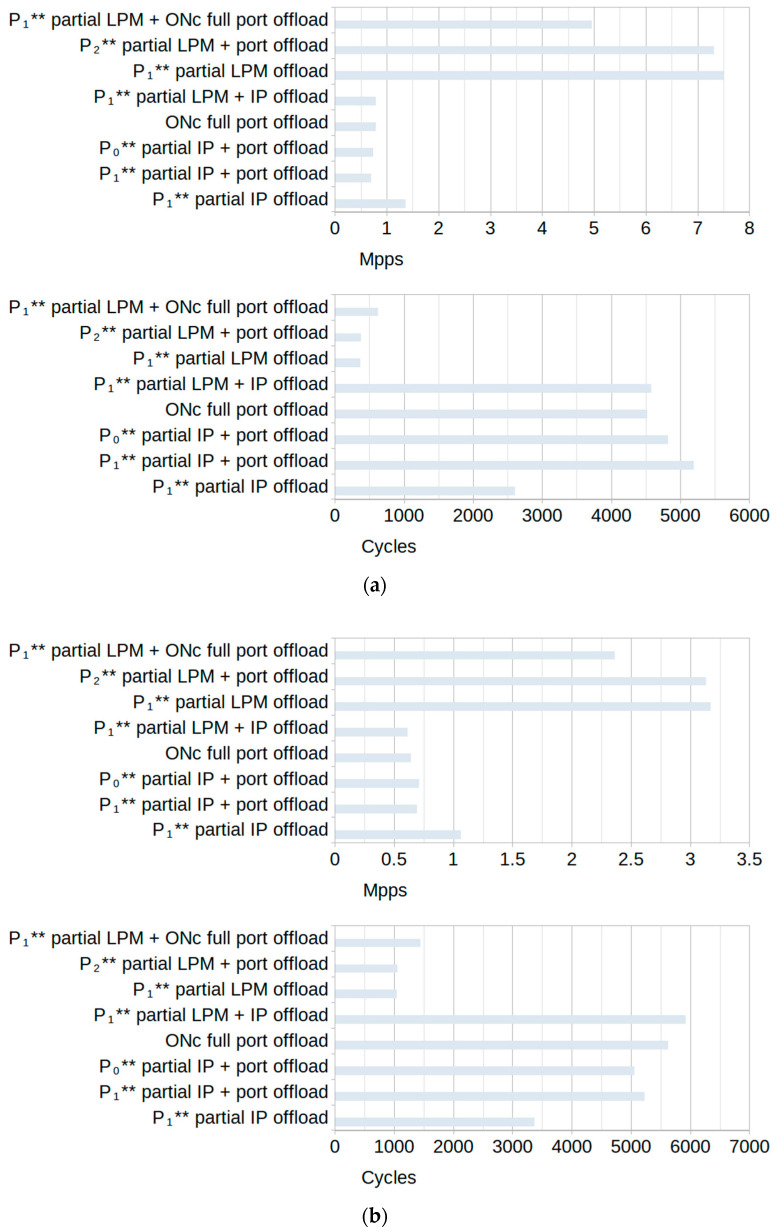
Software-only average filtering throughput and average cycle count of rulesets without *terminating* rules for (**a**) *random* traffic and (**b**) *specific* traffic.

The rulesets used in tests were divided into two groups, depending on the type of rules used in them. One group used only *non-terminating* rules, forcing the software filter to process each of them before forwarding the packet to the egress interface. This ensured that the same number of processing operations were performed for each packet, making the filtering comparable for all of the tests for the same group. The average software-only filtering throughput and cycle count for each type of ruleset in this group are shown in Figure 7.

**Figure 8:**
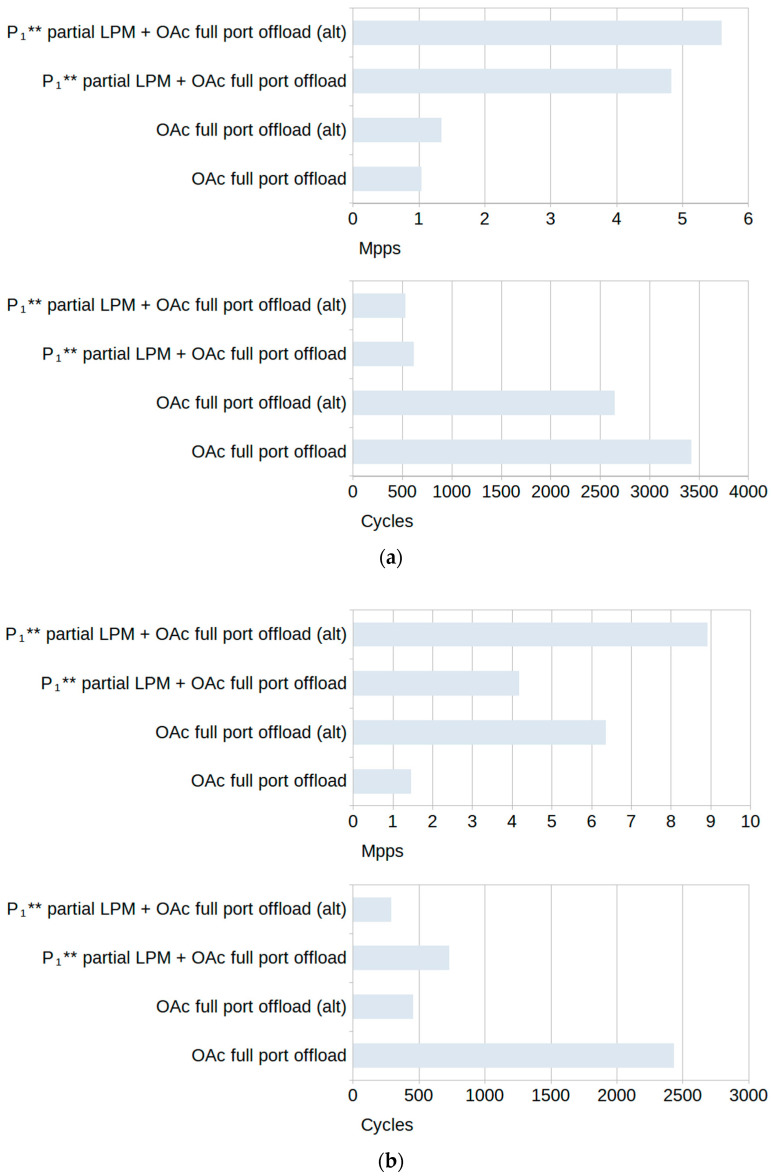
Software-only average filtering throughput and average cycle count of rulesets with *terminating* rules for (**a**) *random* traffic and (**b**) *specific* traffic.

The second group used both *non-terminating* and *terminating* (Accept) rules. If the packet matched the *terminating* rule, it no longer needed to be processed, and so the subsequent rules were not checked. For this reason, the maximum throughput for these tests was slightly higher than the throughput for the tests from the first group of rulesets. The average software-only filtering throughput and cycle count for each type of ruleset in this group are shown in Figure 8.

**Figure 9:**
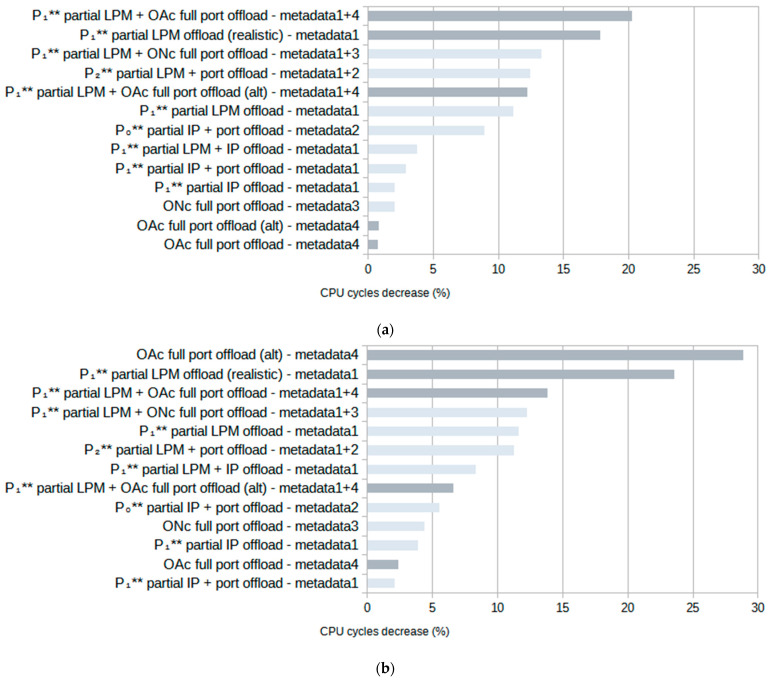
Improvements in average CPU cycle count for (**a**) *random* traffic and (**b**) *specific* traffic. Both types of ruleset are combined in this figure: with *terminating* rules (dark) and without *terminating* rules (light).

Figure 9 illustrates the average CPU cycle count reduction achieved when using metadata for filter offloading, considering both *random* and *specific* traffic. The total throughput increase closely correlates with the CPU cycle decrease, and so it is not shown.

The results in Figure 9 use the metadata notation from Table 3 and show that tests using rulesets with *terminating* rules (*OAc*) show the greatest improvements for both types of traffic. For *random* traffic, the greatest improvement is seen using the ruleset with rules that partially offload the LPM algorithm, combined with rules that fully offload simple port checks to hardware with 20.2% fewer CPU cycles. For *specific* traffic, it is the variation in a ruleset that fully offloads simple port checks to hardware with 28.9% fewer cycles.

The second highest test in both *random* and *specific* traffic cases uses the “realistic” version of the LPM ruleset with rules that can be assumed to be used in a realistic scenario, similar to the rules shown in Figure 4. Using the *P*_1_** partial LPM offloading, it achieved a 17.8% reduction in CPU cycles for *random* and 23.6% for *specific* traffic. Other cases with the highest improvements for both types of traffic are those combining *P*_1_** or *P*_2_** partial LPM offloading with other metadata for an around 10% reduction in CPU cycles.

As previously mentioned, the results shown are all based on tests using metadata pre-generated by the packet generator. To test the hardware part of the hybrid system, i.e., how the system works when the NetFPGA generates the metadata and attaches it to the packets, another set of tests was performed.

**Figure 10:**
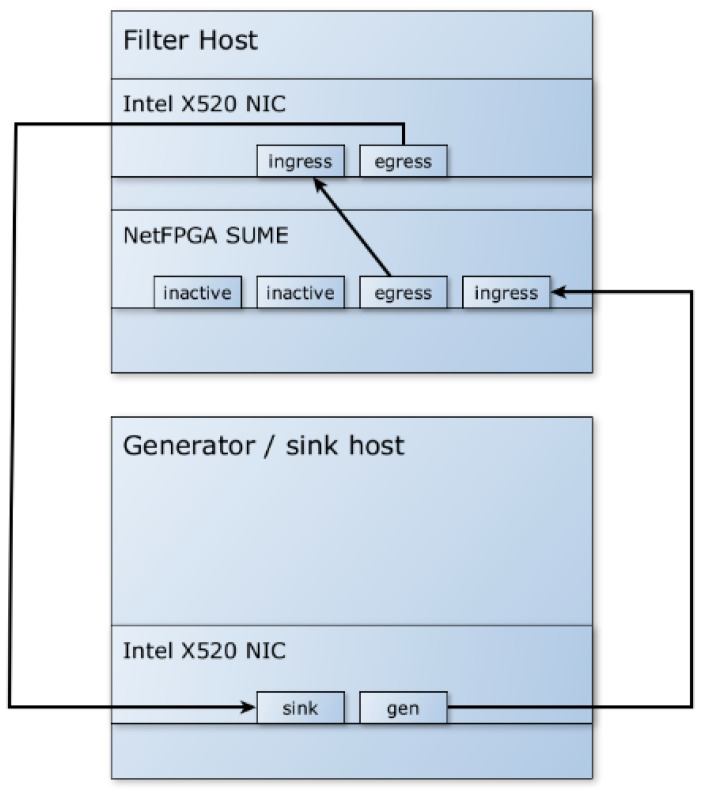
Hybrid hardware/software system testbed.

The test environment (testbed) for the hybrid tests, as shown in Figure 10, was similar to the one used when the hardware part was simulated by the software metadata generator. The traffic generator is connected to the NetFPGA SUME ingress interface, and the SUME egress interface is connected to the software filter ingress interface.

The tests for this hybrid system were performed in the same manner as the simulated tests, except that the packet generator did not have to create metadata and attach it to the packets. For this reason, it was expected that the results of all of the tests performed on the hybrid system would match the results of the simulated hardware. In all of the tests that were performed with the hybrid system, the average results of the hybrid system matched the average results of the simulation almost perfectly. From these results, it can be inferred that it is possible to achieve the same level of improvement over non-offloaded filtering with other types of metadata if specific offload capabilities are implemented in the hardware.

### 4.2. Improving Hybrid Filtering

The extent of improvement in each test may vary depending on the capabilities of the hardware, with performance being further enhanced as more metadata are offloaded to the hardware. Among the different metadata types, *metadata1* requires the fewest and least complex hardware updates to achieve a significant increase in DDoS protection. For example, to increase the total number of available LPM tables for hardware offloading, additional memory needs to be installed in the hardware. The use of this additional memory would not significantly impact the internal FPGA logic and overall system performance. On the other hand, other metadata types require more complicated changes, as parallelism cannot be leveraged as efficiently as in the case of *metadata1*. This could lead to delays and performance degradation, especially when offloading a large number of rules to hardware.

Improving the performance of the filter that uses hardware offloading means finding a balance between the size of the metadata and their usefulness. Responding to changes in the type and volume of traffic is also one of the most important matters to consider when offloading and even beforehand when creating the ruleset. Therefore, in cases where offloading has a negative impact on throughput, it can be bypassed and replaced with a better configuration.

It is worth repeating that all of the tests (including those performed with real hardware offloading) were performed on a system with a single CPU core at a reduced frequency. Moreover, they were performed on a system corresponding to the model shown in Figure 2. The results of a hybrid system without the limitations of this model would certainly be even better.

## 5. Conclusions

In this paper, we presented a datapath model of a high-speed network traffic classifier/filter based on a hybrid hardware/software combination of FPGA and off-the-shelf computer software. The hardware component model comprises a reconfigurable FPGA datapath capable of adapting to runtime packet classification changes in near real-time. On the other hand, the software component is a modified version of the filter used in our previous research, now equipped with additional functionality to receive metadata from the hardware. This integration allows for more efficient packet filtering, leading to improved performance over software-only packet filtering.

We have shown that the hybrid system can achieve filtering in networks with speeds of 10 Gbps by heuristically distributing the workload between the hardware and software components. This is achieved by carefully selecting packet filtering methods and metadata that can be offloaded to the hardware, which optimizes the throughput of the system. To test the system, we developed a method to empirically evaluate the distribution of the workload between the hardware and software components. It bypasses the development of complex hardware implementations by simulating the necessary offloading of hardware to software.

The implemented model shows performance improvements in tests that include both random traffic and traffic specifically designed to simulate DDoS attacks. It is shown that offloading different types of rules to hardware, fully or partially, results in varying performance improvements, with reductions of up to 30% in CPU cycles for certain offloads and rule types. In packet filtering, the use of rules based on LPM offers the advantages of higher throughput and simpler, more manageable rulesets. Therefore, these rulesets are well suited for DDoS protection, and their effectiveness can be further enhanced by hardware offloading.

In addition, the scalability of such a system should be emphasized, because efficient use of the LPM algorithm for IP address lookup means that filtering does not depend on the number of rules, but on the method of offloading parts of the filtering to the hardware. With suitable hardware, it is expected that the improvement in such a system can be maintained at higher speeds.

## Figures and Tables

**Table 1:** Rule offloadability matched with pattern combination types. Multiple repetitions (once or more) of types of patterns combination are marked with ()^+^. Multiple repetitions (zero times or more) of types of patterns are marked with ()*.

Rule Offloadability	Combination of Patterns
*O*	(p_O_)^+^
*P*_0_	(p_N_)^+^(p_O_)^+^
*P*_1_	(p_P_)^+^(p_N_)*
*P*_2_	(p_P_)^+^(p_N_)*(p_O_)^+^
*N*	(p_N_)^+^

**Table 2:** Offload types for rule categories.

Offload Type	Rule Categories
Hardware	*ODn*
Hybrid	*OAc*, *OAn*, *ODc*, *ONc* *P*_0_*Ac*, *P*_0_*An*, *P*_0_*Dc*, *P*_0_*Dn*, *P*_0_*Nc* *P*_1_*Ac*, *P*_1_*An*, *P*_1_*Dc*, *P*_1_*Dn*, *P*_1_*Nc* *P*_2_*Ac*, *P*_2_*An*, *P*_2_*Dc*, *P*_2_*Dn*, *P*_2_*Nc*
Software	*NAc*, *NAn*, *NDc*, *NDn*, *NNc*

**Table 3:** Metadata fields for different rule categories. The ‘*’ character replaces any other attribute.

Rules	Metadata Type			
	*metadata1*	*metadata2*	*metadata3*	*metadata4*
*ODn*	-	-	-	-/x
*OAn*	-	-	-	x
*ONc*	-	-	x	-
*O*[*A*|*D*]*c*	-	-	-	x
*P*_0_ **	-	x	-	-
*P*_1_ **	x	-	-	-
*P*_2_ **	x	x	-	-
N**	-	-	-	-

## Data Availability

All data analyzed during this study are included in this article.
